# Personalized Exposure Assessment: Promising Approaches for Human Environmental Health Research

**DOI:** 10.1289/ehp.7651

**Published:** 2005-03-03

**Authors:** Brenda K. Weis, David Balshaw, John R. Barr, David Brown, Mark Ellisman, Paul Lioy, Gilbert Omenn, John D. Potter, Martyn T. Smith, Lydia Sohn, William A. Suk, Susan Sumner, James Swenberg, David R. Walt, Simon Watkins, Claudia Thompson, Samuel H. Wilson

**Affiliations:** ^1^Division of Extramural Research and Training, National Institute of Environmental Health Sciences, National Institutes of Health, Department of Health and Human Services, Research Triangle Park, North Carolina, USA; ^2^Centers for Disease Control and Prevention, Atlanta, Georgia, USA; ^3^Office of the Director, National Institute of Environmental Health Sciences, National Institutes of Health, Department of Health and Human Services, Research Triangle Park, North Carolina, USA; ^4^Department of Neuroscience, University of California at San Diego, La Jolla, California, USA; ^5^Environmental and Occupational Health Sciences Institute, University of Medicine and Dentistry of New Jersey at Rutgers University, Piscataway, New Jersey, USA; ^6^Schools of Medicine and Public Health, University of Michigan, Ann Arbor, Michigan, USA; ^7^Fred Hutchinson Cancer Research Center, Seattle, Washington, USA; ^8^School of Public Health and; ^9^Department of Mechanical Engineering, University of California at Berkeley, Berkeley, California, USA; ^10^Research Triangle Institute, Research Triangle Park, North Carolina, USA; ^11^Department of Environmental Science and Engineering, University of North Carolina at Chapel Hill, Chapel Hill, North Carolina, USA; ^12^Department of Chemistry, Tufts University, Medford, Massachusetts, USA; ^13^ Department of Cell Biology and Physiology, University of Pittsburgh, Pittsburgh, Pennsylvania, USA

**Keywords:** body burden, epidemiology, exposure, exposure assessment, exposure technology, geographic information systems, GIS, sensors

## Abstract

New technologies and methods for assessing human exposure to chemicals, dietary and lifestyle factors, infectious agents, and other stressors provide an opportunity to extend the range of human health investigations and advance our understanding of the relationship between environmental exposure and disease. An ad hoc Committee on Environmental Exposure Technology Development was convened to identify new technologies and methods for deriving personalized exposure measurements for application to environmental health studies. The committee identified a “toolbox” of methods for measuring external (environmental) and internal (biologic) exposure and assessing human behaviors that influence the likelihood of exposure to environmental agents. The methods use environmental sensors, geographic information systems, biologic sensors, toxicogenomics, and body burden (biologic) measurements. We discuss each of the methods in relation to current use in human health research; specific gaps in the development, validation, and application of the methods are highlighted. We also present a conceptual framework for moving these technologies into use and acceptance by the scientific community. The framework focuses on understanding complex human diseases using an integrated approach to exposure assessment to define particular exposure–disease relationships and the interaction of genetic and environmental factors in disease occurrence. Improved methods for exposure assessment will result in better means of monitoring and targeting intervention and prevention programs.

Well-designed epidemiologic studies are the desired approach for defining the relationships between environmental exposures and human disease. This is partly because human health studies provide the research framework for addressing issues of individual susceptibility to exposure and disease. Furthermore, they are much richer in relevant information than is simple extrapolation from laboratory studies with nonhuman models. This is underscored in recent articles highlighting the importance of designing studies in which interactions between the environment and genetics can be examined to address important health outcomes (Collins 2004; Potter 2004). There is wide agreement in the scientific community that diseases that contribute the greatest public health burden to society result from complex interactions between genetic and environmental factors, such as chemical pollutants, nutrition, lifestyle, infectious agents, and stress (Doll and Peto 1981; Hemminki et al. 2001; World Cancer Research Fund Panel 1997). Environmental factors are an attractive target for disease prevention, especially when susceptible subgroups within the population can be identified. The lack of accurate, quantitative measures of exposure, and information about their relationship to one another and to disease, is the greatest source of uncertainty in epidemiologic studies, limiting the power of such studies to enable definitive conclusions about the association between exposure and disease. New technologies are available for improving exposure assessment in human health investigations and can be exploited in environmental health research, creating a public health strategy for guiding health research and for translating basic research findings into effective prevention, intervention, and treatment efforts.

## Exposure Assessment Methods

The cornerstone of exposure assessment in epidemiologic studies is the development of the exposure metric, the estimate of exposure for each individual of the study population. Ideally, the metric is developed independently for each individual using an actual measurement of exposure that can be validated (Schulte and Perera 1993). Typically, the exposure metric is based on the concentration of specific chemicals, their metabolites, or reaction products in a biologic sample such as blood, urine, or saliva. Obtaining an actual exposure measurement may not be practical when the exposure has occurred in the past and can no longer be detected in a biologic sample. In these instances, the metric is usually developed from environmental monitoring data and chemical transport and fate models using assumptions about the activity patterns and age-specific variables that predict exposure in relation to frequency, duration, and route of entry into the body [Lioy 1990; U.S. Environmental Protection Agency (EPA) 1989). Thus, a theoretical construct is developed for estimating potential exposure to hypothetical or actual individuals of the study population. Uncertainty is an obvious concern when the exposure assessment is derived from a theoretical rather than an evidence-based construct.

**Figure 1:**
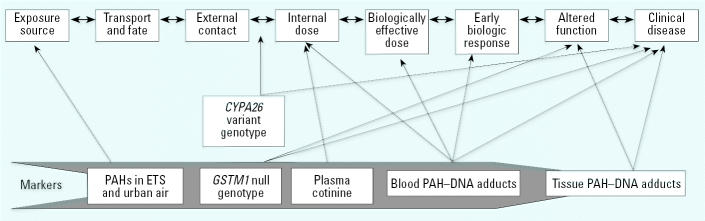
A schematic representation of markers of exposure, response, and susceptibility in the exposure–disease continuum: an example for PAHs and cancer. *CYP2A6,* cytochrome P4502A6 gene; ETS, environmental tobacco smoke; *GSTM1*, glutathione *S*-transferase M1 gene; PAHs, polycyclic aromatic hydrocarbons; Arrows indicate predictability of each marker for exposure or disease in the exposure–disease continuum. Adapted from NRC (1987). PAHs in ETS and urban air are a marker for exposure source. *GSTM1* null genotype and blood PAH–DNA adducts are independent markers of cancer case status (disease) but have a multiplicative effect in combination (Perera et al. 2002; Tang et al. 1995). *GSTM1* null genotype is a predictor of tissue PAH–DNA adducts, which are a marker for altered function (Perera et al. 2002; Rundle et al. 2000; Tang et al. 1995). *CYP2A6* variant is a marker for increased internal dose of nicotine and protective effect on cancer development (Spitz et al. 2005). Plasma cotinine is a marker for internal exposure to ETS but is not correlated with blood PAH–DNA adducts (Mooney et al. 2005). Blood PAH adducts are a marker for PAH/ETS exposure, internal dose, biologically effective dose, early biologic response, and cancer (Mooney et al. 2005; Perera et al. 2002, 2004; Poirier and Beland 1992; Veglia et al. 2003; Whyatt et al. 1998). Tissue PAH–DNA adducts are a marker for altered function and cancer (Rundle et al. 2000).

Epidemiology relies on inference of associations between exposure and response variables. Typically, the measurements of response in epidemiologic studies reflect late-stage end points of morbidity, mortality, body weight decrease, tumor development, and tissue pathology (Bocchetta and Carbone 2004; Maier et al 2004). Defining risk at a late stage in the disease process provides little opportunity to intervene and redirect the outcome. It is clearly more desirable to identify early changes in biologic processes that can serve as predictive markers of exposure, of early effect, or of susceptibility [Committee on Biological Markers of the National Research Council (NRC) 1987]. These components of the proposed continuum between exposure and disease have been described in a number of reports (Lioy 1990; Maier et al. 2004; NRC 1987; Omenn 2002; Perera and Weinstein 1982; Pesch et al. 2004; Schulte 1989; Waters and Fostel 2004). It is important to remember that the distinctions between exposure and response in this continuum are arbitrary. As scientists, we are not able to measure dynamic biologic processes in real time; instead, we must rely on static measurements made at a single or multiple time points. Thus, some markers may represent the event of interest, or may be the event itself, or may be a predictor of the event (Goldstein 1995; Schulte and Perera 1993). This point is illustrated in Figure 1 using data from several human studies evaluating genetic and biologic markers of susceptibility, biologically effective dose, and carcinogenesis from exposure to polycyclic aromatic hydrocarbons (PAHs) in tobacco smoke and urban air (Mooney et al. 2005; Perera et al. 2002, 2004; Poirier and Beland 1992; Rundle et al. 2000; Tang et al. 1995; Veglia et al. 2003). Collectively, the findings illustrate that genetic and biologic markers reflect different biologic events in the overall exposure–disease continuum. The events do not necessarily reflect linearity, although they may be related, nor do they conform to the conventional boundaries for exposure and response. Appropriately, the assessment of exposure and risk should focus on understanding the biologic processes of human disease by defining markers that represent and can link events, both genetic and environmental, in the exposure–disease relationship. In this context, there are multiple approaches for defining the relationship between exposure and disease; some of these will be based on qualitative data that are not readily amenable to conventional dose–response analysis, and each will require a specialized assessment and validation strategy (Rebbeck et al. 2004a; Schulte and Perera 1993).

The biologic response to environmental exposure occurs as a result of complex interactions between multiple genetic, environmental, and behavioral factors, highlighting the importance of defining markers of genetic variation that confer differential functional significance in target cellular pathways. Several programs supported by the National Institute of Environmental Health Sciences (NIEHS), such as the Environmental Genome Project and Children’s Environmental Health Sciences Centers, focus on identifying genetic determinants of susceptibility and the role of gene–environment interactions in disease (Gilliland et al. 2002a, 2002b; Lan et al. 2004; Rollinson et al. 2004; Skibola et al. 2004). For example, a cross-sectional study of benzene-exposed shoe workers (Lan et al. 2004) identified two genetic variants in key metabolizing enzymes, myeloperoxidase and NAD(P)H:quinone oxidoreductase, that influence susceptibility to benzene hematotoxicity, showing a strong gene–dose effect that persisted in workers exposed to benzene at very low levels (< 1 ppm). The findings are particularly robust in relation to previous occupational studies of benzene-induced hematotoxicity because, in this study, personalized exposure monitoring was conducted over a 16-month period and individual air monitoring was linked to specific end points of toxicity. The study highlights the use of personalized exposure and genetic information to define individual susceptibility.

Despite recent advances in genetic susceptibility studies, challenges remain in defining the functional significance of genetic variants and their interaction with environmental factors in biologic systems. In general, health researchers lack reliable, high-throughput, and cost-effective approaches to measure early changes in biologic processes, particularly at the molecular level. The information can provide insight into the biologic and health significance of genetic variants in human populations. Even when information about early molecular and cellular events is available, it is often difficult to interpret in relation to a point of departure from normal physiologic adaptive response to adverse response. At present, the usual information available generally provides limited insight into the relationship between exposure and health risk in individuals or populations.

**Figure 2:**
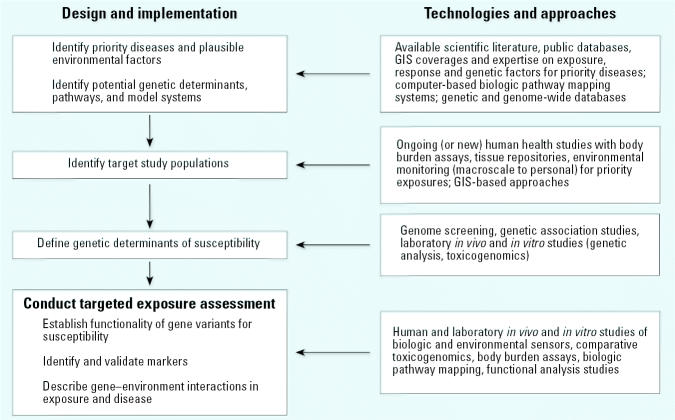
Conceptual strategy for integration of new exposure assessment technologies in human environmental health research.

An ad hoc Committee on Environmental Exposure Technology Development met several times during the summer of 2004 to identify new technologies and methods for improving exposure assessment in human health research. The committee identified a “toolbox” of methods that can be used alone or in combinations to provide information about individual exposure for a variety of exposure scenarios (Lioy 1995). Certain methods within the toolbox, such as environmental sensors and geographic information systems (GIS), can be used to derive information about external environmental exposures and the personal activity patterns that influence the magnitude, frequency, duration, and pathways of exposure. Other methods, such as biologic sensors, toxicogenomics, and body burden assays, can be used to derive measurements of internal biologic exposure. Linking the data sets across multiple scales provides an integrated view of exposure that is needed to define complex exposure–disease relationships and the interplay between genes, environmental factors, and behavior in disease occurrence (Lioy 1995; Maier et al. 2004). We discuss each of these methods in relation to current applicability to human health studies; we also identify specific gaps in the development, validation, and application of the methods. We identified specific activities as first-generation (first 5 years) and second-generation (5–10 years) priorities for moving these technologies into full use and acceptance by the scientific community (Table 1). Finally, the committee developed a conceptual framework for integrating these new technologies in human health studies (Figure 2). The strategy identifies elements of the study design and implementation where new approaches to human-exposure assessment can be incorporated. Implementation of the strategy focuses on common complex human diseases, such as asthma and respiratory disease, neurodegenerative disease, and cancer, each of which represents a significant public health burden to society. Adopting a disease-first approach to exposure assessment allows researchers to take full advantage of new scientific approaches that are currently available in order to advance current knowledge about important diseases (Wilson and Suk, in press). In addition, for each of these outcomes there is substantial evidence of genetic and environmental risk, providing a logical basis for focusing new health research, prevention, and intervention efforts.

## Environmental Exposure Methods

### Environmental sensors.

Environment-sensing devices provide exposure information on a variety of scales, including macro-level exposures in the ambient environment such as from industrial effluents; microscale exposures in the household, workplace, and personal environment; and nanoscale exposures at the points of human contact. Over the past 15 years, there has been significant progress in the development of sensors for monitoring a variety of chemical and biologic agents in the ambient and personal environment (Cui et al. 2001; Georgieva et al. 2002; Haruyama 2003; Svitel et al. 2001). Macroscale technologies such as laser-based and infrared-radiation–based sensors are currently being used for assessing population exposures to sulfur and nitric oxides in industrial-stack effluents. Other microscale sensors, including personal dosimeters, are being used to monitor levels of carbon dioxide, carbon monoxide, volatile organic compounds, pesticides, and PAHs in the workplace, household, and personal environment. The benefits of using microscale over macroscale devices for environmental agents include increased sensitivity for a range of compound classes, decreased volumes of analytic reagents, and increased throughput and potential for multiplexing.

New technologies to assess environmental exposures at the personal level are being conceived, but development is moving slowly. Recent efforts have focused on automated “lab-on-a-chip” sensing devices for detecting environmental agents (Hood et al. 2004). The devices deliver nanoscale volumes of sample to a detection element based on electricity, fluorescence, affinity, or cell function. The sensors can be very small, inexpensive, and easy to use and offer the potential for continuous monitoring on a global or microscale, making them ideal tools for monitoring personal exposure to individual compounds and mixtures.

The use of environmental sensors for large human studies will remain elusive until important issues related to sample handling and control, comprehensiveness of analytic probes, and validation of results are adequately addressed. Targeted efforts by academic researchers and industry are needed, in the short term, to develop sensing devices to accurately and reliably measure high-priority environmental agents, including chemical and biologic agents and complex mixtures, in small-scale projects that have the potential for scalability. This remains an unmet need that was identified in the NRC report on exposure assessment (NRC 1991). Some efforts could build on existing technological developments of countermeasures against bioweapons based on bacteria, viruses, and toxins (Brown 2004). Personal monitors could include global-positioning systems so that the changes in exposure could be tracked as an individual moves through the environment. In aggregate, this could enable researchers to develop data sets that allow mapping and modeling of patterns of exposures across communities.

### GIS technology.

GIS technology is now an increasingly popular tool for developing the exposure metric in epidemiologic studies (Beyea and Hatch 1999; Jarup 2004; Nuckols et al. 2004). The technology is used to create distinct electronic data displays or layers that can be linked spatially and temporally using mathematical modeling systems or tools. The data displays are created by piecing together available environmental, population, and land-use data sets. Many of the available data sets have been generated by government agencies for purposes of national surveillance and environmental regulation. For example, the U.S. EPA and the U.S. Geological Survey (USGS) monitor ambient environmental contaminant levels, and the U.S. Census Bureau collects information about population demographics and land-use patterns. The data sets can provide information about the sources and concentrations of environmental agents at specific monitor points, the nature of the immediate neighborhood, and the characteristics of the study population. The environmental data sets tend to cover very broad geographic scales, such as fixed monitoring stations and contaminant release points for national air and water quality assessment programs (U.S. EPA 2004). Although the data are useful for defining trends in regional environmental quality and assessing compliance with environmental regulations, the scale and resolution may not be appropriate for estimating personal exposures in human studies (Nuckols et al. 2004). Efforts to create more comprehensive environmental data displays and mapping systems that may be used in human health research are under way. For example, the USGS, in collaboration with the NIEHS, has launched the Environmental Mercury Mapping, Modeling and Analysis (EMMMA) website for visualizing the distribution of mercury across geographic, temporal, and biologic scales (EMMMA 2004). The system uses GIS technology to link distinct data sets on mercury levels in environmental media (soil, stream sediments) and multiple fish species and tissues. Web-based mapping tools are used to analyze the mercury data and generate local and national maps of mercury distribution that can be used in human research.

A number of epidemiologic studies have used GIS technology to assess environmental exposures. In many of the early studies, GIS was used to define the study population and to develop the exposure surrogate based on the proximity of the study population to the contaminant source. More recently, GIS has been used in conjunction with computer-based geographic models and analysis tools for predicting contaminant transport and fate, extrapolating between data points to identify potential pathways and routes of exposure, and defining the temporal and spatial distribution of exposure across the study population (Beyea and Hatch 1999; Georgopoulos et al. 1997; Jarup 2004). For example, GIS-based approaches have been used for developing individual metrics for exposure to pesticides, drinking water contaminants, and air pollutants such as nitric oxide, sulfur dioxide, and particulates (Bellander et al. 2001; Kunzli et al. 2005; Nuckols et al. 2004). Only very recently, researchers have been considering the use of GIS to derive personal exposure estimates by linking information about personal activity and behavioral patterns with environmental data (Hellstrom et al. 2004; Jarup 2004; Nuckols et al. 2004). Although no applications of GIS in epidemiologic studies have been reported, several researchers have used GIS with global positioning systems (GPS) to define activity patterns that could conceivably be linked to environmental data for exposure assessment (Elgethun et al. 2003; Nuckols et al. 2004; Phillips et al. 2001). In two studies, a GPS data recorder was used to obtain time–location data that could be linked with information about environmental exposure to pesticides in children (Elgethun et al. 2003) and used to validate exposure information collected by personal diaries (Phillips et al. 2001). A recent report used GIS technology to develop a household-level priority model for childhood lead poisoning in North Carolina (Miranda and Dolinoy 2005). The model linked household location and age with blood lead level and demographics to define potentially at-risk individuals and subpopulations. The model could be expanded to include personal activity, health outcome, and genetic information. Establishing temporal and spatial linkages could be used to assess potential exposure–disease relationships and define genetically susceptible individuals (Miranda and Dolinoy 2005). Information about activity patterns could be collected from study participants using biologic sensing devices and GPS units. Using GIS to spatially integrate personal behavior patterns with environmental data will enable researchers to develop hypotheses about exposure–disease relationships that can be tested in focused studies of potentially at-risk individuals or subgroups of the population. Smaller, focused studies provide an opportunity to use more costly and exploratory technologies, such as environmental or biologic sensors and toxicogenomics, to develop more personalized measures of environmental exposure. In general, environmental or biologic measurements with tight coefficients of technical variation but large ranges of real variation will be most informative. If the environmental or biologic variation is small or small in relation to measurement error, the measurement will have little utility for exposure estimation.

## Biologic Exposure Methods

The greatest impediment to conducting environmental epidemiologic studies is the lack of accurate, quantitative measures of exposure at points of human contact and within the organism. Technologies based on biologic sensors, toxicogenomics, and body burden (biologic) measurements may be useful in human studies to address these critical information gaps.

### Biologic sensors.

A common limitation in exposure assessment is the lack of information about patterns of physical activity and behavior that affect the likelihood of exposure, the frequency and duration of exposure, and the uptake and distribution of environmental agents in the body. Personal dosimetry devices are able to measure individual variables related to activity such as motion, temperature, pressure, energy use, respiratory function, and heart rate (Balbatun et al. 2003; Cao et al. 2004; Jianrong et al. 2004; Kalinowski et al. 2004; Koo et al. 2004; Miljanic et al. 2003; Mo and Smart 2004; Salimi et al. 2004). Many of the devices have been applied successfully in clinical, radiologic, and other occupational and laboratory settings. For application to epidemiology, the technologies require reengineering to combine them into a single small device or set of portable devices that provide readouts that can be integrated over space and time. Wireless tracking devices, global positioning systems, and videography can be incorporated into the sensors, allowing researchers to follow enrolled study participants as they move around. Biologic sensors, some of which may contain tracking devices, are being considered for use by the military in homeland defense (Center for Nanoscale Science and Technology 2004) but have not been integrated into epidemiology study designs. Inclusion of tracking devices in personal-dosimetry tools will enable researchers to link data about external and internal exposures with personal activity patterns. Establishing such linkages provides a basis for developing models for predicting personal biologic exposure based on external measurements that are more readily available in epidemiologic studies.

Biosensors are devices that contain a biologic sensing agent, such as an enzyme, antibody, or microorganism, to detect the presence of a specific analyte in the body. Detection of the analyte produces a biologic change that is converted by a transducer component into a measurable output, such as an electrical or optical signal (Mo and Smart 2004). Biologic sensors hold great promise for improving exposure assessment because many such devices provide rapid, accurate, and quantitative detection and monitoring of a variety of exposures, including mixtures, at a personal level (Bayley and Cremer 2001; Cao et al. 2002; Cui et al. 2001; Culha et al. 2004; Fehr et al. 2005; Haruyama 2003; Jianrong et al. 2004; Mehrvar and Abdi 2004; Salimi et al. 2004; Vo-Dinh 2002).

There has been a renaissance in biologic sensor development over the last several decades. Nanoscale technologies are being proposed for use in medical and basic-research applications (National Cancer Institute 2004; www.biosensors-congress.elsevier.com 2004). Miniature sensors with micro- and even nano-scale dimensions are currently being developed, with many technologies available for laboratory- and research-based measurements. New electrochemical and optical sensors, based on such techniques as surface plasmon resonance, surface enhanced Raman spectroscopy, fluorescence, and microelectrodes, offer promise for personalizing exposure assessment. Engineered materials such as polymers, smart materials, nano- and microstructured materials, and affinity-based reagents such as aptamers, phage-display libraries, and single-chain antibodies have been employed for sensing and offer opportunities for providing integrated analyses for environmental epidemiologic studies. Because sensors have the potential to measure continuously, they provide many opportunities for improving our ability to reduce uncertainties in characterizing human exposure. However, continuous monitoring will yield massive data sets that will require sophisticated database structures and query capabilities, as well as new biostatistical tools for analysis and integration. Consequently, studies incorporating biologic sensing technologies will require appropriate computational tools and support (Porod et al. 2004). With their potentially small dimensions, sensors can be integrated into networks to provide global sensing networks in which continuous spatiotemporal monitoring is achieved. Network development is a complex undertaking, however, and will not be feasible in the near future. Nonetheless, activities needed to develop the networks can be readily piggybacked onto current efforts to address homeland security and public health infrastructure (Brown 2004).

### Toxicogenomics for defining molecular signatures.

Toxicogenomics is a broad field that seeks to define, on a global basis, the levels, activities, regulation, and interaction of genes, mRNA transcripts (transcriptomics), proteins (proteomics), and metabolites (metabolomics) in a biologic sample or system. The molecular signature is derived from the complete data set of mRNA, protein, or metabolite signals from a biologic sample using data-reduction approaches. A useful signature is composed of an ensemble of markers that allows exposures or states to be distinguished with high sensitivity and high specificity (Pepe et al. 2001).

Toxicogenomics methods are evolving at different rates, largely because of trends in scientific discovery, available funding, and ease of platform development and validation. Overall, the experimental methods are approachable, although improvements are still needed to increase sample throughput, quantification, and information yield per sample and to decrease costs. Toxicogenomics-based methods are widely used in laboratory settings to develop biomarkers of exposure, early biologic response, and susceptibility. The approaches have been used for classifying exposures to a variety of chemicals and drugs, for example, hydrazine, 2-bromoethanamine, lead acetate, cadmium, and acetaminophen based on mechanism of action and dose; they have been used for classifying health outcomes for cardiovascular disease and cancer based on disease status and severity (Brindle et al. 2002; Chevalier 2004; Choi et al. 2004; Chung et al. 2002; Coen et al. 2003; Griffin et al. 2001; Hamadeh et al. 2002; Holleman et al. 2004; Holmes et al. 2000, 2001; Kimura et al. 2004; Petricoin et al. 2004a, 2004b; Posadas et al. 2004; Robertson et al. 2000; Tallman et al. 2004; Troyer et al. 2004). Toxicogenomics approaches have been used for predicting the mechanism of action of toxicants and drugs (Gavanagh et al. 2000; German et al. 2003; Phelps et al. 2002; Toraason et al. 2004) and for characterizing the functional significance of genetic polymorphisms (Raamsdonk et al. 2001; Suarez-Merino et al. 2005). The primary basis of classification and discovery in these studies is the molecular signature. The signature itself provides little information about the underlying mechanisms of biologic response. However, once the discriminating elements of the molecular signature are identified, biologic function can be inferred by mapping components to known biologic pathways and verifying functionality in follow-up studies.

Misclassification of exposure and outcome is an important source of bias in epidemiologic studies, and most study designs provide little opportunity to focus on biologic mechanisms underlying the exposure–disease relationship. Toxicogenomics-based methods are being increasingly used in epidemiology and clinical studies, but inclusion is sporadic, primarily due to the lack of readily obtainable, stable, and abundant sample material. Despite the enormous promise of toxicogenomics for advancing our understanding of the relationship between environmental exposure and disease, the challenge has been, and will continue to be, the development of genetic and biologic markers that are predictive of adverse health outcomes in both experimental and human studies.

Toxicogenomics provides an opportunity to move beyond traditional approaches to exposure assessment based on one chemical agent in one environmental medium (air, water, soil) at a time, to a more realistic view of exposure involving multiple exposures, at potentially low environmental concentrations, and multiple biologic response pathways. This comprehensive view of exposure is needed to define complex exposure–disease relationships and the interactions between genetic and environmental factors in human disease. Achieving this goal will require new information and sophisticated modeling capabilities to annotate the components of biologic pathways and to describe their interactions under normal homeostatic conditions and after perturbation by environmental agents. Model development is time consuming, however, and requires a critical mass of appropriate data from human and experimental laboratory studies to be collected and organized. In the short term, molecular signature studies conducted using laboratory animals and human cells lines will be useful for guiding the interpretation of exposure data from epidemiologic studies.

Background levels of expression and variability for mRNA transcripts, proteins, and metabolites in human tissues are currently not known but must be defined if toxicogenomics methods are to be used to assess personal exposures in epidemiologic studies. Expression levels are expected to vary widely because of differences in diet, lifestyle, health status, and genetic predisposition. In addition, expression changes due to low-level environmental exposures may not be distinguishable from baseline, given this inherent variability. Developing background measures of expression for mRNA transcripts, proteins, and metabolites in biosamples will take time and require a critical mass of data. Equally important is the need for technology platforms that produce reliable, quantitative, and stable measurements over time. Standards are needed to assess the quality and reliability of data and to facilitate data sharing and compilation across multiple studies using a variety of technology platforms. Efforts to develop data and technology standards for transcriptomics, proteomics, and metabolomics are under way (Ball et al. 2004; Freeman 2004; Henry 2004; Kaiser 2002; Lindon et al. 2003; Omenn 2004; Orchard et al. 2004; Weis 2005). Preliminary findings suggest that data reproducibility across laboratories is highest when standardized data reporting requirements, technology platforms, and experimental protocols are used (Lindon et al. 2003; Weis 2005; Yauk et al. 2004). These are important considerations for designing epidemiologic studies to ensure that meta-analyses and inferences can be made across other populations.

### Body burden (biologic) measurements.

Assays are available to measure individual body burden or internal dose for a variety of environmental agents, including heavy metals, phthalates, and organic compounds [Barr et al. 2003; Marin et al. 2004; Metcalf and Orloff 2004; National Health and Nutrition Examination Survey (NHANES) 2005; Pirkle et al. 1995]. Most of the assays are based on the quantification of chemicals, their metabolites, or reaction products in blood and urine samples. For most chemicals, the assays produce reliable, quantitative measurements. However, there are several limitations of the available assays. Many assays lack the sensitivity and specificity needed to detect the broad range of compounds present in biologic samples, and many assays have not been validated at background levels in the environment. Most assays can accommodate only moderate sample throughput. Sample collection for blood is invasive and requires clinical supervision and informed consent, which can limit sample collection from infants and children. Modern analytic methods have increased sensitivity, thus requiring smaller sample volumes, which are easier to obtain. Biologic measurements of body burden are difficult to interpret in relation to the biologically effective dose and to early biologic response, but they can provide helpful links to associated health outcome. Population-based surveys such as NHANES, a program of the Centers for Disease Control and Prevention (CDC), provide information about the distribution of many environmental chemicals in the U.S. population (NHANES 2005). Such population estimates are useful for benchmarking measurements of individual exposure in epidemiologic studies but are not designed to provide information about the relative contribution of multiple dietary and lifestyle factors, and the impact of genetic variability and stress on susceptibility. These components of exposure are critical to the understanding of dose in the context of the likelihood of adverse effects and the need for intervention.

A number of innovative, sensitive, and specific methods for measuring internal dose, including biologically effective dose, are currently under development. New methods based on traditional technologies such as chromatography and mass spectrometry are being implemented in the CDC biomonitoring program (Barr et al. 2003). The methods are being used in basic research to identify exposure biomarkers for environmental chemicals based on DNA and protein adducts (Barr et al. 2003; Chen et al. 1996; Perera et al. 2004; Swenberg 2004). Quantification of DNA and protein biomarkers also provides information about the role of genetic polymorphisms in exposure susceptibility for important environmental compounds such as PAHs (Perera et al. 2004; Swenberg 2004). The methods for biologic measurement offer a wide range of sample collection matrices, including hair, saliva, and tissue, and are capable of detecting a myriad of compounds in a single sample. Continued development of these methods is needed to solve problems in sample collection and analysis, sample throughput, and instrument sensitivity.

Additional research is needed to define the functional significance of DNA and protein adducts in human disease processes. Adduct formation occurs naturally at a high frequency in the human genome, making it difficult to define the relative contribution of environmental stressors to overall genomic alteration and to assign a predictive value to specific adduct formation for the risk of developing human disease. Studies are needed to link biologic measurements of body burden or adduct formation to environmental concentrations of exposure and to early biologic responses that are predictive of adverse outcome. Establishing such quantitative links will enable researchers to develop more accurate models for predicting internal dose based on external environmental concentrations and for predicting disease risk based on internal dose. With such improvements, biologic measurements will become an invaluable source of information for personalizing exposure assessment in human health studies.

## A Strategy for the Future

Clearly, a continuum exists between biologic markers of exposure and effect. Each step along the way is an opportunity for prevention through elimination or minimization of exposure (Goldstein 1995) or, in the case of beneficial exposures such as some dietary constituents, augmentation. Realistically, single markers may never provide a definitive answer linking environmental exposure to disease because disease processes involve complex interactions among genes, environmental factors, and behavior. What is needed is a combination of genetic and biologic markers linking critical events in the exposure–disease continuum and a toolbox of methods to derive them. The toolbox should include new experimental technologies, and bioinformatics and statistical tools (Molidor et al. 2003; Rebbeck et al. 2004a), to assess the contribution of multiple genetic variants in multiple biologic pathways and health end points.

As an ad hoc Committee on Environmental Exposure Technology Development, we identified a toolbox of promising new methods, and improvements to existing methods, to personalize exposure assessment in human health research (Table 1). Specific activities needed to enhance technology development for exposure assessment are identified as first generation and second generation. Highest priority is given to activities that *a*) address needed improvements that are readily identifiable and achievable and fill critical gaps in knowledge and *b*) generate information that is high quality, reliable, and stable over time. The toolbox is intended to facilitate technology applications in exposure assessment in the public and private sectors. It is clear that application of new technologies will require multidisciplinary teams of exposure analysts, epidemiologists, clinicians, molecular biologists, toxicologists, statisticians, and bioinformaticians because the new approaches cannot be applied successfully by any one discipline independently.

We developed a conceptual framework for integrating these new technologies in human health research. The framework focuses on common complex human diseases, such as asthma and respiratory disease, neuro-degenerative disease, and cancer, for each of which there is substantial evidence of genetic and environmental risk and each of which represents a significant public health burden. Environmental agents can be used to understand the disease processes by defining the interaction between genes and environmental factors in susceptible populations (Schwartz et al. 2004). This framework combines human and laboratory studies and incorporates new technologies, as appropriate, to answer the biologic questions of interest. For example, recent work by Kiechl et al. (2002) used a prospective population-based survey approach to identify important genetic variants in the toll-like receptor 4 (*tlr4*) that confer differential susceptibility to airway inflammatory response from inhaled bacterial lipopolysaccharides (LPS). As a follow-up, quantitative trait locus (QTL) analysis and microarray-based gene expression analysis were combined in a study of genetic recombinant inbred mice strains with differential susceptibility to inhaled LPS to identify target genes (*n* = 28), in addition to *tlr4*, that may have a causal or modifier role in the innate response to LPS (Cook et al. 2004). Functional genomics approaches can then be used to assess the biologic significance of the target genes and their protein products in biologic pathways of response.

The framework identifies aspects of the study design and implementation where new approaches to exposure assessment can be incorporated to identify genetic variants of susceptibility, link genotype and phenotype data for targeted diseases and exposures, and assess the functional significance of targeted gene variants and their interactions with environmental factors. These aspects of the study design and implementation are presented in Figure 2 and discussed briefly here.

### Identification of priority diseases, plausible environmental factors, genetic determinants, pathways, and model systems.

This identification can be accomplished by reviewing the available scientific literature and publicly available databases of environmental, health, and genetic information. Many of the available data sources are maintained by federal agencies such as the National Institutes of Health (NIH), the U.S. EPA, and the CDC. In addition, academic institutions, hospitals and health care facilities, and industries have developed surveillance programs for specific exposure and health indices in targeted populations. A variety of data sources, such as the GeneSNPs database (Environmental Genome Project 2005) and the International HapMap Project (2005), can be used to identify target gene polymorphisms in human and animal populations. Biologic pathway mapping systems can provide insight into potential biologic processes and research targets for priority diseases. Workshops and meetings can be convened for brainstorming and establishing research priorities. Participants would include representatives from government agencies, academia, and industry who are responsible for environmental and health surveillance, and other scientific experts in exposure assessment, molecular epidemiology, clinical medicine, toxicogenomics, public health, toxicology, and bioinformatics. Workshop participants could define priority diseases and data sources for plausible environmental exposures and genetic susceptibility that are readily available or feasible to obtain.

### Identification of target study populations.

Given the exploratory nature of many new exposure assessment technologies, it is not practical to apply them in all human health studies. One approach to study population selection is to identify existing, well-designed and controlled studies that could benefit from the inclusion of new data to improve the exposure assessment aspect of the study. The NIEHS supports a number of environmental health studies focusing on identifying genetic and environmental risk factors and gene–environment interactions in asthma and respiratory disease, neurodegenerative disease, and cancers. Other NIH institutes, the CDC, the U.S. EPA, and other agencies have ongoing studies that may be appropriate. The NHANES program periodically seeks recommendations of new assays for its studies. Highest priority should be given to studies with clearly defined disease outcomes, quantifiable environmental exposures that may be plausibly related to the disease, and an accessible study population. Inclusion of new exposure assessment technologies into these ongoing studies, in particular to derive personalized exposure measurements for individuals or subpopulations at greatest risk of exposure or disease, provides a cost-effective approach to explore the practicality of their implementation and the usefulness of the data they generate. Specific study populations or subpopulations for which body burden measurements, personal monitoring data, and tissue repositories are available or can be readily obtained are particularly attractive candidates.

In addition, new study populations can be identified using global screening tools such as GIS-based technologies to identify specific sub-populations with unusually high rates of the disease or potentially elevated exposures for the disease. The NIEHS and U.S. EPA support a number of investigators who are currently using GIS-based approaches as part of ongoing research projects (Kunzli et al. 2005; Miranda and Dolinoy 2005). Application of GIS-based technologies, together with information about personal activity patterns for the study participants, can be used to identify and target specific subpopulations for in-depth personalized assessment of exposure.

### Determinants of genetic variability and susceptibility.

Genotyping can be applied to human studies to identify genetic variants that may predispose individuals to environmental exposure or disease. Genetic linkage and association studies have been used to identify potential susceptibility genes for a number of outcomes, including asthma and chronic obstructive pulmonary disease (Meyers et al 2004; Sharma et al. 2004), inflammatory response to inhaled bacterial pathogens and atherogenesis (Kiechl et al. 2002), acute myeloid leukemia (Rollinson et al. 2004), non-Hodgkin lymphoma (Skibola et al. 2004), and lung cancer (Liu et al. 2004). Some of these studies involved genotyping families to define disease-related genes that co-segregate with DNA markers (Meyers et al. 2004; Sharma et al. 2004). Well-established familial cohorts are an excellent resource for conducting gene discovery studies, especially for complex disorders where disease subtypes (e.g., type I vs. type II diabetes, breast cancer) can be discriminated within families (Merikangas 2003). Both family and twin studies have been useful for determining the relative contribution of genetic and environmental factors in disease occurrence, although the findings may not be generalizable to other populations. Population-based studies are useful for identifying the distribution of newly identified polymorphisms in the population, in particular, susceptibility genes that have low population frequencies. Knowledge of population genetic structure may provide insight into the functional relevance of a genetic variant on a disease trait (Rebbeck et al. 2004a). Public databases containing information about single nucleotide polymorphisms (SNPs) in human populations can be used to identify target gene variants for further study. Once genetic susceptibility genes are identified, other approaches, such as nested case–control studies for specific susceptibility genotypes, may help define environmental factors that contribute to disease risk.

### Targeted exposure assessment.

Targeted exposure assessment is needed to identify valid genetic and biologic markers, determine the functional significance of genetic variants, and describe gene–environment interactions in disease. New technologies can be used to define markers of external environmental exposure based on human activity patterns and personal monitoring, and markers of internal biologic dose and response based on body burden measurement, sensors, and toxicogenomics. For many complex diseases, environmental risk factors are not known; application of new approaches provides an opportunity to identify important environmental and behavioral risk factors for disease. Exposure information generated using new approaches should be considered complementary to information collected by study questionnaire or survey, in particular regarding occupational, dietary, and lifestyle factors. To the extent possible, quantitative linkages between environmental data and personal exposure measurements should be established as a basis for developing predictive models for exposure assessment. Integrating data from these new approaches into the study design and data analysis phases will require appropriate rigor of data and sample collection and validation that is intrinsic to the best epidemiologic and clinical research. In addition, improvements in analytic, statistical, and bioinformatics tools will be needed to support the integration of molecular, clinical, and epidemiologic data in human studies (Molidor et al. 2003).

Concurrent studies in appropriate animal models and primary human cell cultures should be considered for developing and validating genetic and biologic markers, establishing the functional significance of candidate genetic variants, and gaining mechanistic insight into gene–environmental interactions in human disease. Many model organisms are not as genetically diverse as are humans but have orthologous genes and biologic pathways that are represented in humans. Comparative studies in model systems with shared genes, functions, and pathways provide the greatest opportunity to define biologically relevant responses to environmental exposures and the impact of genetic variation on that response in humans (Schwartz et al. 2004).

New technologies for personalizing exposure assessment will benefit the scientific and regulatory community by providing range-finding and sensitivity matrices for specific methods, developing baseline data on important environmental factors, and improving the results of exposure-model simulations. Efforts to address genetic or genomic variation alone will have little value in personalizing human exposure assessment unless there are effective linkages with information about environmental and behavioral variables that affect the likelihood of exposure and risk. Therefore, future studies will require that personal genetic information be linked with estimated or measured personal exposure data, while ensuring that individual privacy is protected.

## Correction

In the last paragraph of “Exposure Assessment Methods,” the authors added information about the benefit of adopting a disease-first approach to exposure assessment.

## Figures and Tables

**Table 1:** A toolbox of promising exposure assessment technologies and activities for integration in human environmental health research.

Technology	First-generation activities	Second-generation activities
All technologies	Identify priority diseases, plausible environmental exposure factors (including dietary and lifestyle factors, infectious agents), genetic determinants, biologic pathways, and model systems	Develop background ranges and study population distribution of parameters for priority environmental exposures, response parameters, and genetic variants
	Identify and review available scientific literature and databases in government, academia, and industry	
	Convene a workshop of experts to establish research priorities.	
Environmental sensors	Develop and validate *in vitro* sensors for detecting and quantifying priority environmental exposures	Develop multiplexed sensors for continuous monitoring of priority environmental exposures
	Develop analytic tools and approaches to link environmental data across multiple scales, from macroenvironmental to personal	Develop integrated sensor networks
GIS technology	Select priority environmental and population data sets and develop GIS displays	Initiate studies using environmental and biologic sensors and other exposure assessment methods to generate GIS displays for individualized exposure assessment in targeted studies
	Develop and apply modeling and mapping tools to link environmental and personal exposure data to identify at-risk populations	
Biologic sensors	Develop wearable personal sensors for monitoring activity patterns	Develop deployable *in vivo* (microscale and nanoscale) sensors for monitoring biologic responses to priority exposures
	Develop data management and analytic to support biologic sensing devices	
	Develop *in vitro* diagnostic sensors for monitoring early biologic responses to priority environmental factors	Develop sensor networks
Toxicogenomics*^a^*	Select preferred technology platforms	Conduct human and animal studies to validate molecular signatures as markers of exposure, response and susceptibility, and define biologic response pathways for priority exposures and responses
	Develop data and technology standards	
	Develop improved methods of sample preparation and analysis (throughput)	
	Initiate human and animal studies to develop molecular signatures as markers of exposure, response and susceptibility, and define disease processes	
Body burden assays	Develop and apply assays to quantify priority exposures in biologic samples	Develop and apply new methods to assess biologically effective doses for priority exposures and mixtures
	Improve methods of sample preparation and analysis	Conduct studies to link body burden with biologically effective dose and environ- mental levels for priority exposures
	Improve sample matrix selection, and assay sensitivity and selectivity	

New methods, and improvements to existing methods, to personalize exposure assessment in human health research. Specific activities needed to enhance technology development for exposure assessment are identified as first generation (0–5 years from today) and second generation (5–10 years from today).

a Refers to global analysis of genes, gene expression transcripts (transcriptomics), proteins (proteomics), and metabolites (metabolomics).
